# Common Laws Driving the Success in Show Business

**DOI:** 10.1155/2020/8842221

**Published:** 2020-07-10

**Authors:** Chong Wu, Zhenan Feng, Jiangbin Zheng, Houwang Zhang

**Affiliations:** ^1^Department of Electrical Engineering, City University of Hong Kong, Kowloon, Hong Kong; ^2^School of Automation, China University of Geosciences, Wuhan 430074, China; ^3^School of Informatics, Xiamen University, Xiamen 361005, China

## Abstract

In this paper, we want to find out whether gender bias will affect the success and whether there are some common laws driving the success in show business. We design an experiment, set the gender and productivity of an actor or actress in a certain period as the independent variables, and introduce deep learning techniques to do the prediction of success, extract the latent features, and understand the data we use. Three models have been trained: the first one is trained by the data of an actor, the second one is trained by the data of an actress, and the third one is trained by the mixed data. Three benchmark models are constructed with the same conditions. The experiment results show that our models are more general and accurate than benchmarks. An interesting finding is that the models trained by the data of an actor/actress only achieve similar performance on the data of another gender without performance loss. It shows that the gender bias is weakly related to success. Through the visualization of the feature maps in the embedding space, we see that prediction models have learned some common laws although they are trained by different data. Using the above findings, a more general and accurate model to predict the success in show business can be built.

## 1. Introduction

“Do I need to change a job?” is one of the major concerns to most actors and actresses since the show business is really competitive [1]. Matthew effect [2] or the so-called “rich-get-richer” phenomenon is proved to exist in the show business which demonstrates the scarcity of the resources [1]. Luck is proved to be a key element in driving the success [3]. It is well known that the effect of rich-get-richer is quite arbitrary and unpredictable [4]. Hence, most actors and actresses will meet a problem of avoiding the famine and building a sustainable career in acting [1]. Some studies have found that boosting productivity is a key metric to evaluate the success of an actor or actress, and it can be more of a network effect [5, 6] than a consequence of acting skills; in other words, success is not highly related to the acting skills [1]. And, some studies show the relationship between the dynamic collaboration network and success [7]: success is a collective phenomenon [8]. Startup network is proved to have predictive power in show business [9]. And, future success can be predicted by monitoring the behavior of a small set of individuals [10]. To study the law of success, a great deal of work has been done [11–19].

Recently, a study shows that the success in show business is predictable and uses a heuristic threshold-based binary classifier to achieve an accuracy up to 85% [1]. In their study, they find a strong gender bias in the waiting time statistics, the location of annus mirabilis, and the career length distribution of these data. However, we have some questions here: Whether gender bias is one of the key elements driving the success? Can we find some common laws driving the success in show business? Since we want to build a general prediction model, the common laws which determine the growth and the shape of the series are more important than the differences.

To solve our questions, we design this study. The data we use are collected from the International Movie Database (IMDb), http://www.imdb.com in [1]. It consists of millions of profile sequences of actors and actresses from the birth of the film in 1888 up to the present day [1]. Each sequence records the yearly time series of credited jobs over the entire working life of the actor or actress [1]. We just consider the number of credited jobs regardless of the impact of the work, the screen time, and so on, which is the same as in [1]. The original feature space is a non-Euclidean space. We must to do the representation learning to map these features to a Euclidean space. To do this, we construct a deep model which consists of an encoder and a classifier. Since gender is an independent variable in our experiment, we train three models: (1) MAO, (2) MAE, and (3) MM. They all have the same structure but are trained by different datasets (MAO is trained by the data of an actor, MAE is trained by the data of an actress, and MM is trained by the mixed data). Our problem can be reconstructed like follows: (1) if MAO can achieve nondegradation performance on the data of an actress like MAE and MAE can achieve nondegradation performance on the data of an actor like MAO, then it can be proved that there are common features in the series which are unrelated to the gender. (2) If MM can achieve similar and nonsuperior performance against MAO and MAE, then these features which have gender bias are not dominative features in this prediction problem; that is to say, gender bias may cause some differences into the resource allocation, but it is weakly related to success. The contributions of this paper can be concluded as follows:

1. We found that there are some common laws/features driving the success in show business by extracting and understanding the data.
2. Using these common features, a more general prediction model with an accuracy up to 90% can be built.
3. Our experiment shows that gender bias is weakly related to success despite a recent study which shows that it affects strongly the waiting time statistics, the location of annus mirabilis, the career length distribution, etc.

## 2. Materials and Methods

### 2.1. Data

The data we use consist of the careers of 1,512,472 actors and 896,029 actresses from 1888 up to 2016 and are collected from the International Movie Database (IMDb) http://www.imdb.com. Each career is viewed as a profile sequence: the yearly time series of acting jobs in films or TV series over the entire working life of the actor or actress [1]. We refer to [1] and relax their selection constraint to select the sequences of actors and actresses with working lives *L* ≥ 5 years, and the number of credited jobs in the annus mirabilis (AM) is ≥ 5. The sequences obtained by some more relaxed cutoffs are too short to be analyzed, and they are considered as the outliers and not included in the experiment. Then, the subset we use consists of 37896 (2.51%) sequences of actors and 22025 (2.46%) sequences of actresses which is larger than the data used in the prediction model in [1]. We divide this subset into several groups for experiment: (1) Group 1: the data of an actor with AM ≥ 5 and *L* ≥ 20, including 21994 sequences; (2) Group 2: the data of an actress with AM ≥ 5 and *L* ≥ 20, including 9034 sequences; (3) Group 3: the data of an actor with AM ≥ 5.5 ≤ *L* < 20, including 15902 sequences; (4) Group 4: the data of an actress with AM ≥ 5.5 ≤ *L* < 20, including 12991 sequences. Group 1 and Group 2 can be considered as some very successful actors which are used to train the prediction model mainly. Group 3 and Group 4 can be considered as some actors who are not very successful, and they might need a prediction model more than previous groups, and these data will be used to test the prediction model.

### 2.2. Data Preprocessing

**Figure 1:**
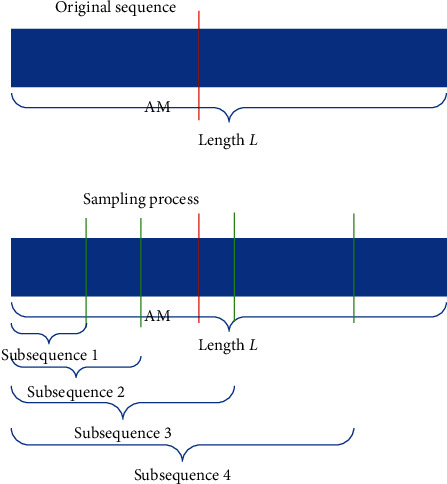
The process of subsequence generation.

To do an early prediction, we need to do some preprocessing on the data before training the model. At first, we refer to [1] to truncate each sequence into several subsequences or called subcareer series. For each sequence, we randomly sample several subsequences with a sampling rate *n*. The subsequences which are sampled before the annus mirabilis are regarded as class 1. The subsequences which are sampled after the annus mirabilis are regarded as class 2. Hence, it is a binary classification problem. The aim of this sampling is to get some samples of class 1 since we only have the entire working life of the actor or actress. An example of the sampling process with a sampling rate *r* = 4 is shown in Figure 1. NatComm19 uses the following function [1] to transfer these subsequences to scalars for the training:

(1) $$\begin{array}{c} D\left({\bar{w}}_{T}\right)=-\sum_{y=1}^{T-1}\min\left(0,{\bar{w}}_{y+1}-{\bar{w}}_{y}\right), \end{array}$$

where ${\bar{w}}_{T}$ is the number of credited jobs at year *T* and *T* is the length of the subsequence.

The above transformation will lose some information like the increasing or decreasing trend. In this paper, we revise equation (1) as follows to get a new sequence and not a scalar which will protect these information:

(2) $$\begin{array}{c} D\left({\bar{w}}_{T}\right)=-\sum_{y=1}^{k-1}\min\left(0,{\bar{w}}_{y+1}-{\bar{w}}_{y}\right). \end{array}$$

Then, we use the new sequence *D* to train the model.

Since gender is an independent variable, we construct three prediction models which will be trained by different subsets of the whole data. The details of separation of training data and test data for each model are shown in Table 1.

### 2.3. Prediction Model

**Figure 2:**
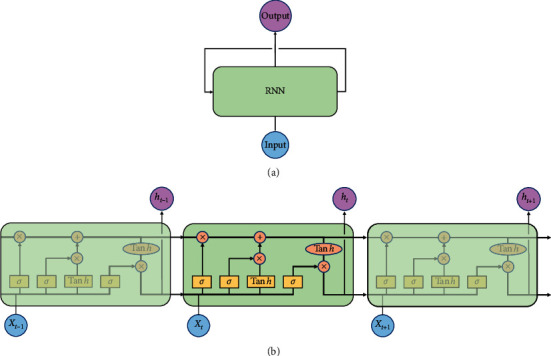
The structure of RNN and details of the LSTM unit.

**Figure 3:**
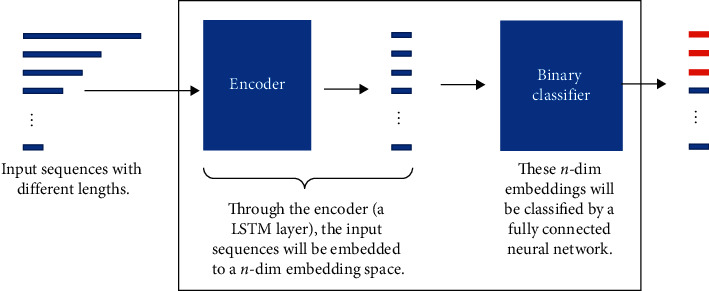
The workflow of our model. It has an end-to-end structure and can be divided into two parts: (1) encoder; (2) binary classifier. The encoder of our model is a single LSTM layer which is used to embed different sequences to an *n*-dim embedding space. The binary classifier is a fully connected neural network.

**Figure 4:**
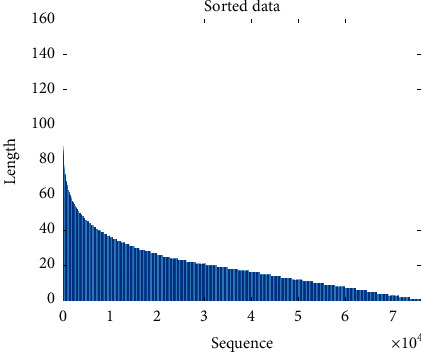
Sequences sorted by the sequence length.

Recurrent neural network (RNN) or long short-term memory (LSTM) [20, 21] is powerful to solve the time series prediction problem with sequential data. Compared to the standard feedforward neural network, RNN is a kind of neural networks which is as the feedback connections (memory), as shown in Figure 2. It can process not only single data points, but also the entire sequences of data. For example, LSTM is applied in some tasks such as speech recognition [22], sign language translation [23], object cosegmentation [24, 25], and airport passenger management [26]. Hence, here, we use RNN with LSTM units to build an end-to-end prediction model, where the LSTM unit is composed of a cell, an input gate, an output gate, and a forget gate.  Figure 3 shows the structure of our model. Sequentially, our model can be divided into two parts: (1) encoder; (2) binary classifier. The encoder consists of an LSTM layer with 30 hidden units and outputs at the last time step. And, the classifier consists of a fully connected layer, a softmax layer, and a classification layer with the cross entropy as the loss function. Our model is trained in a supervised fashion, on a set of training sequences, using an optimization algorithm, gradient descent. Since sequences have different lengths as shown in Figure 4, the feature space of these sequences is a non-Euclidean space. It is difficult to train a classifier in this feature space. Hence, each input sequence will be embedded by the encoder to a Euclidean space using the following transformation:

(3) $$\begin{array}{c} f:D⟶H, \end{array}$$

where *H* is an *n*-dim sequence. Through the encoder, the dimension of the feature is also reduced. Then, the following loss function is minimized to get the optimized parameters:

(4) $$\begin{array}{c} L\left(C,\hat{C}\right)=-C\log\left(\hat{C}\;\right)-\left(1-C\right)\log\left(1-\hat{C}\;\right), \end{array}$$

where *C* is the real label and $\hat{C}$ is the label predicted by the classifier.

In the process of forward propagation, LSTM does not simply compute a weighted sum of the input signal. It applies a nonlinear function. For each *j*-th LSTM unit, it maintains a memory *c*_*i*_^*j*^ at time *j* and an output gate weight *o*_*i*_^*j*^. Then, the output *h*_*i*_^*j*^ is

(5) $$\begin{array}{c} h_{i}^{j}=o_{i}^{j}\tan h\left(c_{i}^{j}\right). \end{array}$$

The memory cell *c*_*t*_^*j*^ is updated by partially forgetting the existing memory and adding a new memory content *c*_*t*_^*j*′^:

(6) $$\begin{array}{c} c_{t}^{j}=f_{t}^{j}c_{t-1}^{j}+p_{t}^{j}c_{t}^{j^{\prime }}, \end{array}$$

where *f*_*t*_^*j*^ is the weight of the forget gate and *p*_*t*_^*j*^ is the weight of the input gate.

The details of each layer's configuration are shown in Table 2. The training settings for the prediction model: max epoch is set to 15, size of the minibatch is set to 100, optimizer is Adam, and gradient threshold is set to 1. More complex models like the models with deep layers and the models with complex structures (biLSTM) have also been tested, but there is no obvious performance improvement. That is to say, these are all fairly “off the shelf ” classifiers. Since simpler is better, we just use the simplest model to show the results.

## 3. Results

Table 3 [Table tab4]–5 show the comparison between our model and a recent study NatComm19 [1] on the test data. MM_ours denotes the prediction model trained by the mixed data of an actor and actress, MAO_ours denotes the prediction model trained by the data of an actor only, and MAE_ours denotes the prediction model trained by the data of an actress only. MM_NatComm19 denotes the model of NatComm19 [1] trained by the mixed data of an actor and actress, and the learned threshold *d* = 6.1523; MAO_NatComm19 denotes the model of NatComm19 [1] trained by the data of an actor only, and the learned threshold *d* = 6.9580; and MAE_NatComm19 denotes the model of NatComm19 [1] trained by the data of an actress only, and the learned threshold *d* = 5.6640. All models are trained on the training data with a cutoff value (AM ≥ 5, *L* ≥ 20). We can see that our models outperform NatComm19 in terms of all quantity metrics in all subsets of the test data. Our models are more general than NatComm19 and can still maintain the performance on the new data (AM ≥ 5, 5 ≥ *L* < 20 and AM ≥ 10, 5 ≥ *L* < 20 and AM ≥ 15, 5 ≥ *L* < 20), whereas the performance of three models of NatComm19 degrades to near the baseline. The details of the baseline model can be found in [1]. There is almost no difference between the performance of our three models. And, interestingly, the difference between the performance of the three models of NatComm19 can also be ignored.

## 4. Discussion

**Figure 5:**
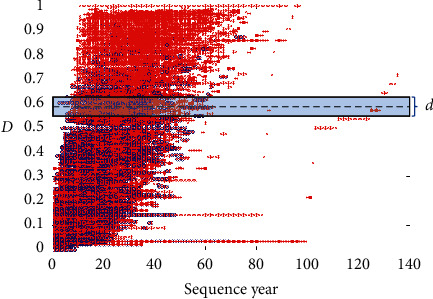
The workflow of the model in NatComm19 [1]. *d* is a scalar threshold which is learnable. The target of this model is to get an optimal *d* to separate two classes in the original feature space.

**Figure 6:**
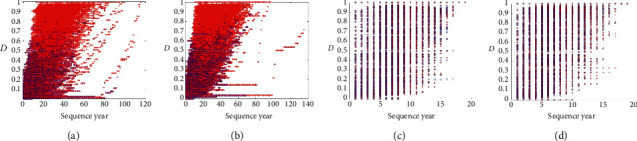
Feature maps of the original feature space. *Note*. There are a few outliers (sequences with a length over 100). It is caused by a few films that in some sense exist but have not been released. Since they are so rare and are the correct data, they are also considered as in [1]: (a) actor, AM ≥ 5, *L* ≥ 20; (b) actress, AM ≥ 5, *L* ≥ 20; (c) actor, AM ≥ 5.5 ≤ *L* < 20; (d) actress, AM ≥ 5.5 ≤ *L* < 20.

**Figure 7:**
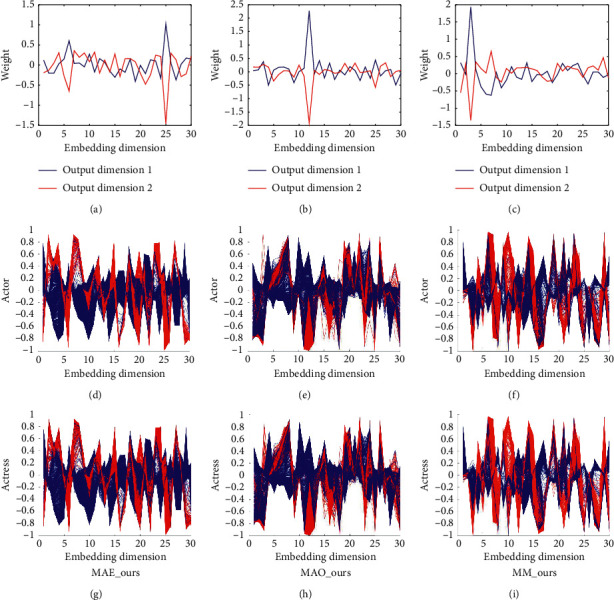
Feature maps of the testing data of an actor and actress in the embedding space obtained by different models. Blue line denotes class 1, and red line denotes class 2. It can be seen that the curves of different datasets show the same distribution and shape in the same embedding space. And, the boundary between two classes is clearer than the original feature space. Although it seems that the embedding spaces of different models are different, they are actually equivalent because they are different approximations of the global optimum obtained by the neural network. And, the curves of each feature's weight show that there is one feature dominating the classification. Note that it is like the eigen decomposition. Hence, the order of these weights has no meaning. And, the dominative feature of each model shows a similar floating range, and there is a clear boundary between two classes in this feature. It further proves that three models have learned a similar feature.

Two MAE models (MAE_ours and MAE_NatComm19) can achieve similar results compared to two MAO models (MAO_ours and MAO_NatComm19) on the test data of an actor. Similarly, two MAO models (MAO_ours and MAO_NatComm19) can also achieve similar results compared to two MAE models (MAE_oursandMAE_NatComm19) on the test data of an actress. The case of MAE_ours and MAO_ours shows that our models can learn some common features that are used to classify. Since the model of NatComm19 uses a learnable threshold to classify the original feature space as shown in Figure 5, the case of MAE_NatComm19 and MAO_NatComm19 shows that the distribution and the shape of the original feature space of the data of an actor and the data of an actress are similar just as shown in Figure 6. MM_ours achieves similar and nonsuperior results compared to MAE_ours and MAO_ours, and MM_NatComm19 also achieves similar and nonsuperior results compared to MAE_NatComm19 and MAO_NatComm19. It shows that these features which have gender bias are not dominative features in this prediction problem; that is to say, gender bias may cause some differences in some aspects like resource allocation, but it is weakly related to success. To further validate our conclusion, we visualize the embedding space in Figure 7. It seems that three models learn some different features. But, it was caused by the randomness of the neural network, and the order of these features has no meaning because it is like the eigen decomposition. From the weight of each embedding feature which is obtained in the fully connected layer, we can see that most of these embedding features are unimportant. And interestingly, all three models have only one dominative feature. The floating range of the corresponding feature in three models is also similar [−1,  *s*], where *s* is a positive scalar. We can believe that they have learned a similar feature that is used to classify.

## 5. Conclusion

In this paper, we design a data-driven research to find out whether the gender bias is a key element and try to find some common laws/features driving the success in show business. The experiment results show that there are some common features between the success of an actor and the success of an actress. And, gender bias is weakly related to the success. We use this property to build a general model to predict the success in show business. Compared to the benchmark, the improvement of the model is obvious. In the future, we plan to do a further research on whether gender bias is a key element and try to find some common laws driving the success in other fields.

## Figures and Tables

**Table 1:** The details of training data and test data for each model.

		Training data including validation data	Test data
Model 1: MAO_ours		70% data of an actor (AM ≥ 5, *L* ≥ 20), the sampling rate of subsequence generation: *n* = 6; the total number of subsequences in the training set: 6^*∗*^0.7^*∗*^21994 = 92374	30% data of an actor (AM ≥ 5, *L* ≥ 20), the sampling rate of subsequence generation: *n* = 6; 100% data of an actor (AM ≥ 5, 5 = < *L* < 20), the sampling rate of subsequence generation: *n* = 5; 100% data of an actress (AM ≥ 5, *L* ≥ 20), the sampling rate of subsequence generation: *n* = 12; 100% data of an actress (AM ≥ 5, 5 = < *L* < 20), the sampling rate of subsequence generation: *n* = 5; the total number of subsequences in the test set: 6^*∗*^0.3^*∗*^21994 + 5^*∗*^1^*∗*^15902 + 12^*∗*^1^*∗*^9034 + 5^*∗*^1^*∗*^12991 = 292462

Model 2: MAE_ours		70% data of an actress (AM ≥ 5, *L* ≥ 20), the sampling rate of subsequence generation: *n* = 12; the total number of subsequences in the training set: 12^*∗*^0.7^*∗*^9034 = 75885	30% data of an actress (AM ≥ 5, *L* ≥ 20), the sampling rate of subsequence generation: *n* = 12; 100% data of an actress (AM ≥ 5, 5 = < *L* < 20), the sampling rate of subsequence generation: *n* = 5; 100% data of an actor (AM ≥ 5, *L* ≥ 20), the sampling rate of subsequence generation: *n* = 6; 100% data of an actor (AM ≥ 5, 5 = < *L* < 20), the sampling rate of subsequence generation: *n* = 5; the total number of subsequences in the test set: 12^*∗*^0.3^*∗*^9034 + 5^*∗*^1^*∗*^12991 + 6^*∗*^1^*∗*^21994 + 5^*∗*^1^*∗*^15902 = 308951

Model 3: MM_ours		70% mixed data of an actor and actress (AM ≥ 5, *L* ≥ 20), the sampling rate of subsequence generation: *n* = 3 for the data of an actor in the mixed data, *n* = 6 for the data of an actress in the mixed data; the total number of subsequences in the training set: 3^*∗*^0.7^*∗*^21994 + 6^*∗*^0.7^*∗*^9034 = 46187 + 37942 = 84129	30% mixed data of an actor and actress (AM ≥ 5, *L* ≥ 20), the sampling rate of subsequence generation: *n* = 3 for the data of an actor in the mixed data, *n* = 6 for the data of an actress in the mixed data; 100% data of an actress (AM ≥ 5, 5 = < *L* < 20), the sampling rate of subsequence generation: *n* = 5; 100% data of an actor (AM ≥ 5, 5 = < *L* < 20), the sampling rate of subsequence generation: *n* = 5; the total number of subsequences in the test set: 3^*∗*^0.3^*∗*^21994 + 6^*∗*^0.3^*∗*^9034 + 5^*∗*^1^*∗*^12991 + 5^*∗*^1^*∗*^15902 = 180520

The validation data are included in the training data. *Note*. MM_ours denotes the prediction model trained by the mixed data of an actor and actress, MAO_ours denotes the prediction model trained by the data of an actor only, and MAE_ours denotes the prediction model trained by the data of an actress only.

**Table 2:** The details of each layer's configuration in our model.

Layer's name	Input size	Output size	No. of hidden units
Sequence input	1	1	30
LSTM	1	—	
Fully connected layer	30	2	
Softmax layer	2	2	
Classification layer	2	2	

**Table 3:** Performance comparison of our methods and a recent study NatComm19 [1] in the prediction of the AM on the subset (AM ≥ 5) of the test data.

	Actor	Actress	Actor	Actress
	L ≥ 20, AM ≥ 5	*L* ≥ 20, AM ≥ 5	5 = < *L* < 20, AM ≥ 5	5 = < *L* < 20, AM ≥ 5
*C*1 : *C*2	0.8074	0.6136	0.7888	0.5655
Baseline accuracy	0.6702	0.7221	0.7034	0.7487

MM_ours				
*F*1 score	0.9102	0.9262	0.8891	0.9173
Precision	0.8866	0.9079	0.8570	0.9037
Recall	0.9350	0.9452	0.9237	0.9313
Accuracy	0.8978	0.9067	0.8702	0.8917

MAO_ours				
*F*1 score	0.9082	0.9254	0.9045	0.9272
Precision	0.9010	0.9267	0.8845	0.9203
Recall	0.9156	0.9241	0.9254	0.9341
Accuracy	0.8992	0.9077	0.8897	0.9048

MAE_ours				
*F*1 score	0.9104	0.9268	0.9021	0.9265
Precision	0.8958	0.9203	0.8537	0.8966
Recall	0.9255	0.9334	0.9564	0.9584
Accuracy	0.8992	0.9087	0.8828	0.9020

NatComm19MM				
*F*1 score	0.7956	0.7878	0.7442	0.7436
Precision	0.8930	0.8346	0.6092	0.6074
Recall	0.7174	0.7459	0.9562	0.9585
Accuracy	0.8338	0.8453	0.7100	0.7099

NatComm19MAO				
*F*1 score	0.7942	0.7872	0.7457	0.7438
Precision	0.8902	0.8347	0.6103	0.6075
Recall	0.7169	0.7448	0.9582	0.9588
Accuracy	0.8332	0.8464	0.7116	0.7111

NatComm19MAE				
*F*1 score	0.7707	0.7770	0.7766	0.7409
Precision	0.9176	0.8803	0.6630	0.6057
Recall	0.6643	0.6954	0.9371	0.9540
Accuracy	0.8238	0.8474	0.7622	0.7591

MM_ours denotes the prediction model trained by the mixed data of an actor and actress; MAO_ours denotes the prediction model trained by the data of an actor only; MAE_ours denotes the prediction model trained by the data of an actress only; MM_NatComm19 denotes the model of NatComm19 [1] trained by the mixed data of an actor and actress, and the learned threshold *d* = 6.1523; MAO_NatComm19 denotes the model of NatComm19 [1] trained by the data of an actor, and the learned threshold *d* = 6.9580; MAE_NatComm19 denotes the model of NatComm19 [1] trained by the data of an actress, and the learned threshold *d* = 5.6640.

**Table 4 tab4:** Performance comparison of our methods and a recent study NatComm19 [1] in the prediction of the AM on the subset (AM ≥ 10) of the test data.

	Actor	Actress	Actor	Actress
	*L* ≥ 20, AM ≥ 10	*L* ≥ 20, AM ≥ 10	5 = < *L* < 20, AM ≥ 10	5 = < *L* < 20, AM ≥ 10
*C*1 : *C*2	0.6481	0.4169	0.6053	0.3348
Baseline accuracy	0.7275	0.7968	0.7668	0.8173

MM_ours				
*F*1 score	0.9409	0.9591	0.9202	0.9530
Precision	0.9355	0.9612	0.9418	0.9780
Recall	0.9463	0.9571	0.8995	0.9293
Accuracy	0.9279	0.9422	0.9024	0.9313

MAO_ours				
*F*1 score	0.9389	0.9557	0.9276	0.9563
Precision	0.9551	0.9729	0.9538	0.9836
Recall	0.9232	0.9391	0.9029	0.9306
Accuracy	0.9270	0.9387	0.9118	0.9359

MAE_ours				
*F*1 score	0.9396	0.9559	0.9377	0.9643
Precision	0.9414	0.9664	0.9338	0.9761
Recall	0.9378	0.9457	0.9415	0.9528
Accuracy	0.9264	0.9386	0.9217	0.9467

NatComm19MM				
*F*1 score	0.7688	0.8008	0.8114	0.7607
Precision	0.9299	0.8879	0.7321	0.6371
Recall	0.6552	0.7292	0.9101	0.9439
Accuracy	0.8460	0.8916	0.8367	0.8478

NatComm19MAO				
*F*1 score	0.7681	0.7989	0.8085	0.7559
Precision	0.9280	0.8841	0.7250	0.6297
Recall	0.6552	0.7287	0.9137	0.9453
Accuracy	0.8453	0.8909	0.8371	0.8449

NatComm19MAE				
*F*1 score	0.7377	0.7790	0.8127	0.7616
Precision	0.9373	0.8956	0.7518	0.6431
Recall	0.6082	0.6892	0.8843	0.9337
Accuracy	0.8330	0.8823	0.8429	0.8502

MM_ours denotes the prediction model trained by the mixed data of an actor and actress; MAO_ours denotes the prediction model trained by the data of an actor only; MAE_ours denotes the prediction model trained by the data of an actress only; MM_NatComm19 denotes the model of NatComm19 [1] trained by the mixed data of an actor and actress, and the learned threshold *d* = 6.1523; MAO_NatComm19 denotes the model of NatComm19 [1] trained by the data of an actor, and the learned threshold *d* = 6.9580; MAE_NatComm19 denotes the model of NatComm19 [1] trained by the data of an actress, and the learned threshold *d* = 5.6640.

**Table 5:** Performance comparison of our methods and a recent study NatComm19 [1] in the prediction of the AM on the subset (AM ≥ 15) of the test data.

	Actor	Actress	Actor	Actress
	*L* ≥ 20, AM ≥ 15	*L* ≥ 20, AM ≥ 15	5 = < *L* < 20, AM ≥ 15	5 = < *L* < 20, AM ≥ 15
*C*1 : *C*2	0.5271	0.3253	0.6292	0.3429
Baseline accuracy	0.7683	0.8439	0.7940	0.8467

MM_ours				
*F*1 score	0.9563	0.9725	0.9236	0.9583
Precision	0.9600	0.9756	0.9548	0.9883
Recall	0.9527	0.9694	0.8945	0.9301
Accuracy	0.9434	0.9584	0.9021	0.9358

MAO_ours				
*F*1 score	0.9533	0.9697	0.9336	0.9618
Precision	0.9750	0.9832	0.9692	0.9934
Recall	0.9326	0.9566	0.9005	0.9322
Accuracy	0.9401	0.9548	0.9159	0.9414

MAE_ours				
*F*1 score	0.9560	0.9710	0.9340	0.9638
Precision	0.9632	0.9794	0.9306	0.9758
Recall	0.9489	0.9627	0.9375	0.9520
Accuracy	0.9425	0.9562	0.9179	0.9458

NatComm19MM				
*F*1 score	0.7647	0.7990	0.8161	0.7425
Precision	0.9303	0.8555	0.7581	0.6161
Recall	0.6492	0.7495	0.8837	0.9340
Accuracy	0.8600	0.9099	0.8593	0.8614

NatComm19MAO				
*F*1 score	0.7608	0.8043	0.7978	0.7588
Precision	0.9230	0.8660	0.7282	0.6345
Recall	0.6470	0.7508	0.8822	0.9437
Accuracy	0.8610	0.9096	0.8491	0.8639
NatComm19MAE				
*F*1 score	0.7361	0.7883	0.7954	0.7552
Precision	0.9268	0.8586	0.7455	0.6391
Recall	0.6105	0.7286	0.8525	0.9230
Accuracy	0.8530	0.9059	0.8489	0.8671

MM_ours denotes the prediction model trained by the mixed data of an actor and actress; MAO_ours denotes the prediction model trained by the data of an actor only; MAE_ours denotes the prediction model trained by the data of an actress only; MM_NatComm19 denotes the model of NatComm19 [1] trained by the mixed data of an actor and actress, and the learned threshold *d* = 6.1523; MAO_NatComm19 denotes the model of NatComm19 [1] trained by the data of an actor, and the learned threshold *d* = 6.9580; MAE_NatComm19 denotes the model of NatComm19 [1] trained by the data of an actress, and the learned threshold *d* = 5.6640.

## Data Availability

The data used in this study can be accessed at https://doi.org/10.17605/OSF.IO/NDTA3.
